# Pruritus and Neuropsychiatric Symptoms Among Patients with Darier Disease—An Overlooked and Interconnected Challenge

**DOI:** 10.3390/jcm14061818

**Published:** 2025-03-08

**Authors:** Grace Xiong, Muskaan Sachdeva, Gil Yosipovitch, Michael Ziv, Roni P. Dodiuk-Gad

**Affiliations:** 1 Michael G. DeGroote School of Medicine, McMaster University, Hamilton, ON L8S 4L8, Canada; grace.xiong@medportal.ca; 2 Division of Dermatology, Department of Medicine, University of Toronto, Toronto, ON M5S 1A1, Canada; 3 Miami Itch Centre, Dr. Phillip Frost Department of Dermatology and Cutaneous Surgery, University of Miami, Leonard M. Miller School of Medicine, Coral Gables, FL 33136, USA; yosipog@gmail.com; 4 Department of Dermatology, Emek Medical Centre, Rappaport Faculty of Medicine, Technion—Institute of Technology, Haifa 1834111, Israel

**Keywords:** genodermatoses, Darier disease, pruritus, psychological health

## Abstract

**(1) Background:** Darier disease (DD) is a rare autosomal dominant disorder caused by mutations in ATP2A2, a gene that encodes the sarco(endo)plasmic reticulum calcium-ATPase 2 enzyme, which disrupts calcium homeostasis in keratinocytes. Pruritus, a frequently overlooked symptom in DD, can lead to physical and emotional complications, especially in patients with DD who are genetically predisposed to psychiatric comorbidities. **(2) Methods:** This study aimed to analyze pruritus and other related symptoms in patients with DD and explore their correlation with neuropsychiatric conditions, psychological challenges, disease severity, and body surface area (BSA) involvement through a retrospective review of a tertiary center. **(3) Results:** Data from 76 patients (equal gender distribution, mean age 44 years) revealed a prevalence of pruritus of 90.8%, surpassing symptoms such as pain (34.3%) and malodor (43.4%). Burning sensations due to DD lesions were significantly correlated with the diagnosis of comorbid neuropsychiatric conditions (*p* = 0.047) and psychiatric medication use (*p* = 0.019). While pruritus correlated with disease severity and %BSA involvement, the findings were not statistically significant. Patients reporting pruritus had a significantly higher Dermatology Life Quality Index symptom score (2.4 ± 1.0), which is defined as the presence of itch, soreness, pain, or stinging, than those who did not (1.5 ± 0.6), indicating accurate symptom reporting. **(4) Conclusions:** In conclusion, a striking majority of patients with DD experience pruritus, with higher prevalence among those with neuropsychiatric challenges, severe Darier disease, and greater %BSA skin involvement. Clinicians should recognize pruritus as a key therapeutic target and adopt comprehensive treatment approaches that both address the neuropsychiatric comorbidities and the added psychological burden of pruritus in patients with DD.

## 1. Introduction

Darier disease (DD) is a rare, autosomal dominant genetic disorder characterized by abnormalities in epidermal keratinization, presenting as keratotic papules and plaques primarily in seborrheic areas [1]. This condition is caused by a mutation in the ATP2A2 gene, which encodes the sarco-endoplasmic reticulum calcium ATPase (SERCA2) pump and leads to disruptions in keratinocyte calcium homeostasis [1]. The resultant calcium dysregulation can result in defective calcium signaling and altered epidermal differentiation, causing defective adhesion of keratinocytes and the characteristic skin lesions seen in DD [2]. In addition to skin manifestations, DD can present with a variety of symptoms affecting the nails, such as V-shaped notches, ridging, splitting, and longitudinal streaks, or eyes, such as blepharitis and keratoconjunctivitis sicca [1,3,4]. Mucous membranes may also be involved, with symptoms including cobblestone lesions, gingival hypertrophy, xerostomia, and parotid gland obstruction [1,4]. Patients with DD may also experience comorbid neuropsychiatric conditions, such as anxiety and epilepsy, as well as other systemic conditions, including diabetes and heart failure [5,6,7].

Among the numerous cutaneous and systemic symptoms that patients with DD frequently experience, pruritus is one of the most under-evaluated. Pruritus significantly impacts patients with DD, both physically and emotionally. Physically, pruritus can induce complications as the compulsion to scratch may result in excoriations, aggravate skin lesions, and increase susceptibility to secondary infections [1]. The psychosocial consequences of pruritus have been recognized in conditions such as atopic dermatitis and prurigo nodularis [6,7]. However, in DD, pruritus imposes a further emotional challenge as nearly half of DD patients experience psychiatric comorbidities [8]. Various studies found that individuals with DD exhibit a higher lifetime prevalence of mood disorders, anxiety disorders, psychotic disorders, and neurodevelopmental disorders compared to the general population [8,9]. Emerging research has highlighted the pleiotropic role of ATP2A2 gene mutations in the pathogenesis of neuropsychiatric conditions in DD [8,9]. This suggests that there is an inherent genetic predisposition to psychiatric comorbidities. Therefore, patients with DD face the combined emotional impact of genetically predisposed neuropsychiatric symptoms and the added psychological burden of pruritus. This challenging interplay imposes a unique clinical scenario that has yet to be evaluated. 

This study aims to analyze pruritus and other related symptoms in patients with DD and to explore their correlation with neuropsychiatric symptoms, disease severity, and body surface area involvement.

## 2. Materials and Methods

### 2.1. Study Design

Our study is a retrospective analysis of information from a pre-existing database of DD patients collected in a clinical study. In that study, three methods of data collection were used: (1) a comprehensive physical examination; (2) quality of life assessment, including the Dermatology Life Quality Index (DLQI) questionnaire (Cardiff University, Cardiff, UK) [10]; (3) structured clinical interviews using a clinical research form (CRF) (see Supplemental Material S1). Some of the database was analyzed and summarized in previous publications [8,11,12,13,14].

The information analyzed in the current study was obtained from the aforementioned database. Specifically, data on pruritus and neuropsychiatric symptoms were collected through the DLQI questionnaire and Sections “E” and “F” of the CRF. The CRF was completed during physician-guided patient interviews and physical examinations.

### 2.2. Data Collection and Analysis

Descriptive statistics were used to summarize patient characteristics, including proportions, means, and standard deviations, where appropriate. From Section E of the CRF, the following symptoms were evaluated: itch, tingling, pain, burning, discomfort, and malodor. From Section F of the CRF, the presence of anxiety, depression, suicidal ideation, suicide attempts, and other psychological challenges were examined. Dysesthetic and neuropsychiatric symptoms were assessed as binary outcomes, specifically yes/no depending on whether patients reported these symptoms. The association between symptoms reported on the CRF and disease severity was assessed using Chi-square or Fisher’s exact tests. Further analysis was pursued to assess the correlation between symptoms reported on the CRF and the percentage of body surface area (%BSA) affected. This was conducted using Wilcoxon two-sample tests. Finally, the correlation between dysesthetic symptoms and neuropsychiatric symptoms was assessed with Chi-square or Fisher’s exact tests. A *p* value of <0.05 was considered statistically significant.

Data were collected from the first question of the DLQI, where a score of 1 indicates “not at all” itchy, sore, painful, or stinging, and a score of 4 indicates “very much” itchy, sore, painful, or stinging. This was defined as the “DLQI symptom score” in the present study. Intra-patient reliability was assessed with the Wilcoxon two-sample test, by comparing the average DLQI symptom score between patients who reported itch, tingling, or pain on the CRF and patients who did not.

## 3. Results

### 3.1. Patient Characteristics

A total of 76 patients were included. The cohort had an equal gender distribution, and a mean age of 44.0 ± 15.5 years (range: 19–88). The ethnic distribution consisted of patients from Jewish and Caucasian backgrounds (90%) and other Caucasian backgrounds (10%). Patients had a mean body mass index of 28.3 ± 5.4 (range: 18–40), and 21% had a history of smoking.

### 3.2. Disease Characteristics

Disease severity was assessed according to Sakuntabhai et al. [15], with patients being classified as mild (33%), moderate (49%), or severe (18%). Sixty-seven (88%) patients had a family history of DD, supporting its genetic component. The patients had a mean %BSA involvement of 20.5 ± 14.7% (range: 0.2–70). A majority of patients (67%) were not being medically followed for their DD at the time of study.

### 3.3. Outcomes

**Figure 1:**
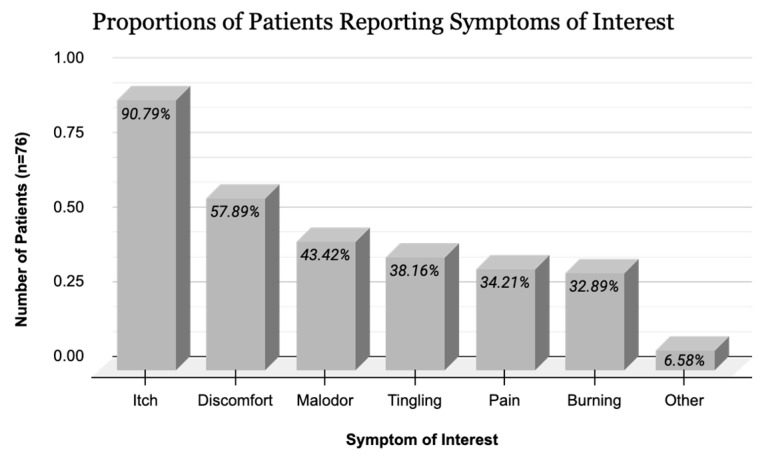
Proportions of patients reporting symptoms of interest.

**Figure 2:**
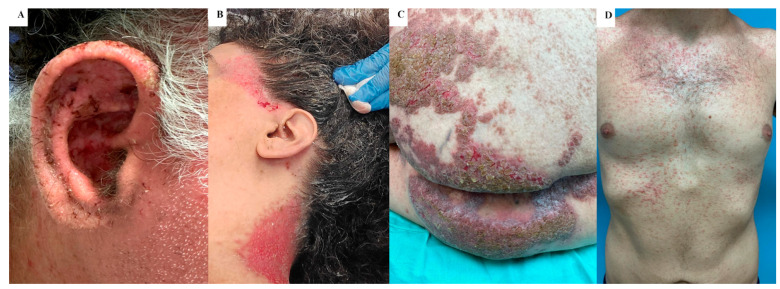
Various pruritic skin lesions in patients with Darier Disease: (**A**) ear, (**B**) temple and neck, (**C**) buttocks, (**D**) chest.

Patients overwhelmingly reported pruritus (90.8%) compared to other symptoms such as pain (34.3%), malodor (43.4%), and discomfort (57.9%). Self-reported symptoms from the CRF are summarized in Figure 1. Various types and locations of DD lesions were found to be pruritic (Figure 2).

A larger proportion of patients with moderate or severe disease reported itch than patients with mild disease, with nearly all patients with severe (92.3%) and moderate disease (97.3%) reporting itch. Itch was also associated with a higher %BSA involvement in patients with DD, with patients experiencing itch having a mean %BSA of 21.2% compared to a mean %BSA of 12.8% in patients without itch. However, these associations were not statistically significant. The results pertaining to disease severity and %BSA involvement are summarized in Table 1 and Table 2, respectively.

The mean DLQI symptom score of patients reporting itch, tingling, or pain in the CRF [2.4, 95% CI (3.4, 1.4)] was significantly higher than that of patients who did not [1.5, 95% CI (2.1, 0.9), *p* < 0.05], as depicted in Table 3. This further suggests that outcomes reported on the CRF are accurate and reliable when compared to a validated scale. Although the definitions of the dysesthetic symptoms in the DLQI and CRF are not identical (itch, pain, soreness, and stinging in the DLQI compared to itch, pain, and tingling in the CRF), we chose to compare them to assess potential correlations between responses on the two forms since patients may struggle to distinguish between itch and other dysesthetic symptoms.

As we previously reported [8], this cohort had a high prevalence of neuropsychiatric symptoms.

When examining the relationship between neuropsychiatric symptoms and dysesthetic symptoms, we found that burning was significantly associated with both past and current use of psychiatric medications (*p* = 0.019) and a diagnosis of various psychiatric disorders (*p* = 0.047). Further associations are depicted in Table 4. Itch was not found to be significantly associated with the assessed neuropsychiatric symptoms and diagnoses.

## 4. Discussion

### 4.1. Summary

Our study found that a striking 90.8% of patients with DD experience pruritus, with a higher prevalence of pruritus among those with more severe disease and a greater %BSA involvement. In addition to pruritus, other dysesthetic symptoms such as pain, tingling, burning, and discomfort, in addition to malodor, were frequently reported in our cohort. These findings highlight the multifaceted nature of sensory disturbances in DD. However, the underlying pathophysiology of pruritus among patients with DD remains poorly understood, with limited research available on this topic.

The existing literature on pruritus in DD primarily comprises case reports and small case series [16,17,18]. A few studies have sought to explore the impact of pruritus in DD. Rogner et al. [19] identified pruritus as the most distressing symptom in DD, with 80.4% prioritizing itch relief as the primary therapeutic target in a cohort of 46 patients. Similarly, Burge et al. [4] reported that 88% of their cohort of 163 DD patients experienced pruritus. Other smaller cohorts have also substantiated pruritus as the most common symptom in patients with DD [20]. Recent research suggests that pruritus in DD may result from the release of pro-inflammatory and pruritogenic cytokines triggered by factors such as ultraviolet light, heat exposure, and secondary infections [21]. In addition, ATP2A2 gene mutations have recently been implicated in Grover’s disease, one of the most pruritic skin conditions [22], suggesting a possible genetic mechanism behind itch in DD.

### 4.2. Darier Disease and Neuropsychiatric Conditions

Patients in our cohort reported a variety of neuropsychiatric conditions and psychological challenges, including anxiety, depression, mood disorders, psychotic disorders, and personality disorders [8]. This aligns with existing literature documenting the higher lifetime prevalence of neuropsychiatric disorders in individuals with DD compared to the general population, including mood-related conditions such as depression and anxiety, bipolar disorder, migraines, epilepsy, neurodegenerative disorders, and various learning disabilities [9,13,23,24,25,26]. Additionally, as individuals with DD have also been shown to exhibit alarmingly high rates of suicidality [8,9], comprehensive management strategies that address the psychological components of the disease are paramount.

While the psychological sequelae of DD are logically, in part, reactive to physical impairments such as pruritus, emerging research has highlighted the heterotrophic role of ATP2A2 gene mutations in the development of neuropsychiatric conditions in DD, implying an inherent genetic predisposition to psychiatric comorbidities [8,9,23]. Specifically, mutations in the ATP2A2 gene result in severe impairment of SERCA2 activity, which is highly expressed in the central nervous system [15,22,24]. In our study, burning sensations were found to be significantly associated with neuropsychiatric diagnoses and psychiatric medication use in DD patients. This interesting phenomenon needs to be further explored.

The consequent dysregulation of calcium homeostasis predisposes patients to various neuropsychiatric conditions [15,22,24]. Calcium is essential to the regulation of many neural processes, including neurotransmission, synaptic activity and plasticity, gene expression, and neuronal metabolism and survival [27]. Calcium dysregulation can result in an imbalance between calcium influx and efflux [27]. Calcium overload, caused by the overactivation of calcium channels such as voltage-gated calcium channels and N-methyl-D-aspartate (NMDA) receptors, can cause cell death [27]. Similarly, dysfunction in calcium pumps or exchangers prevents efficient calcium clearance and activates stress pathways in the endoplasmic reticulum, leading to energy deficits that further exacerbate cellular harm [27]. Furthermore, studies suggest that calcium plays a critical role in not only neural survival, but also neural development. Transient elevations in intracellular calcium, known as calcium transients, ref. [28] have been implicated in neuron proliferation, migration, and differentiation [29]. In vivo imaging studies have shown that neuronal subtypes, such as sensory or motor neurons, each have unique patterns of calcium transients that support axon growth [30]. This makes intact calcium signaling essential for the development of diverse neuronal subtypes and, subsequently, complex neural circuitry [29].

Calcium signaling dysregulation, which contributes to the loss or dysfunction of neuronal diversity, is a key factor in the pathophysiology of many neuropsychiatric disorders. In Alzheimer’s disease, disruptions to calcium homeostasis contribute to synaptic failure, memory loss, and cognitive dysfunction [31]. In Parkinson’s disease, dysregulated calcium signaling impacts the pacemaking activity of dopaminergic neurons, leading to motor and cognitive symptoms [32]. Long-term calcium dysregulation further leads to the excitotoxicity seen in other neurodegenerative disorders such as Huntington’s disease and amyotrophic lateral sclerosis [33]. Schizophrenia and bipolar disorder have also been linked to genetic mutations that disrupt calcium signaling pathways crucial for mood regulation [34]. Other mood disorders, such as depression, have similarly been linked to calcium imbalances that impede serotonin pathways [35]. Calcium dysregulation has been associated with both monogenic and inherited autism spectrum disorders, with various mutations, such as in the CACNA1C gene, impairing neurodevelopment [36]. Overall, there is strong evidence supporting the association between calcium-related cell death or dysfunction causing loss of neuronal diversity and various neuropsychiatric disorders.

This provides a molecular basis for neuropsychiatric manifestations of DD that are independent of the burden caused by physical symptoms and driven by genetic factors. This genetic predisposition is assumed to underlie the frequent psychiatric comorbidities observed in this patient population. Notably, one genetic and familial analysis of 57 individuals with DD found a high prevalence of psychiatric phenotypes [8]. Interestingly, the same study identified variations in the psychiatric manifestations among patients with identical ATP2A2 mutations [8]. These findings suggest that the development of neuropsychiatric comorbidities in DD may involve factors beyond the ATP2A2 mutation and point to a multifactorial mechanism of pathogenesis including interactions between many genetic and environmental factors. Recent studies have begun to examine the association between mechanisms of cell dysfunction in DD and neuropsychiatric comorbidities [37]. This may lead to individualized treatment algorithms in the future based on genetic, molecular, and clinical phenotypes.

Furthermore, the following arguments, as described below, imply that genetic and environmental effectors play a dominant role in the neuropsychiatric manifestations of DD and that the trigger for these associated disorders cannot be attributed solely to reactive phenomena. First, in various studies, disease severity did not correlate with psychological morbidity [9,12]. Second, the incidence of neurodevelopmental disorders such as epilepsy and learning disabilities in DD indicates involvement of the central nervous system [8,13,22]. Finally, if neuropsychiatric symptoms were purely reactive to skin symptoms, they would typically emerge later in life, following the onset of DD which typically occurs around adolescence. However, many patients exhibit neuropsychological deficiencies early in life which either precede or coincide with dermatologic presentation [9,13]. All of the above reinforces the notion of a complex pathogenesis of DD with a genetically mediated component.

### 4.3. Pruritus and Darier Disease

As highlighted by this report, the role of pruritus in DD has yet to be comprehensively assessed. Moreover, the precise mechanisms driving pruritus in DD and its potential role in the exacerbation of genetically predisposed neuropsychiatric conditions remain unknown. In this report, we wish to emphasize not only the high prevalence of pruritus among patients with DD, but also the distinct clinical context in which these patients frequently suffer from pre-existing emotional disturbances, thereby impacting their ability to manage pruritus. This interplay, commonly known as the mind–skin connection, is well documented, illustrating how psychological stress can exacerbate the perception of itch in inflammatory skin conditions [38].

In patients with DD, psychological challenges are exacerbated by the need to cope with the disease’s signs and symptoms [8,9]. Pruritus, a prominent symptom among patients with DD, is a complex sensory experience that profoundly affects psychological well-being [38]. Pruritus is not merely a physical sensation, but a multifactorial experience involving both sensory and emotional components that make it especially difficult to endure [6,39]. The distress associated with pruritus is amplified by its chronicity, intensity, and unpredictability, which has been shown to disrupt sleep, daily functioning, and overall quality of life [6,39].

The physiology of pruritus involves complex neural pathways and signaling molecules. Peripheral sensory neurons in the skin, known as pruriceptors, respond to pruritogens, the itch cytokine, to transmit signals to the spinal cord and brain [39]. Recent research has highlighted the crucial role of calcium signaling in the transmission and modulation of itch. Calcium ions (Ca^2+^) are fundamental to neural signaling and, thus, the perception of sensations such as pruritus [40]. Long-term dysregulation of calcium signaling may impact the sensitivity or stimulation thresholds of pruriceptors, thereby affecting the frequency or intensity of the itch response [41]. Studies have shown the role of calcium-permeable ion channels including TRPV1 (transient receptor potential vanilloid 1), TRPA1 (transient receptor potential ankyrin 1), and more recently, TRPV4 (transient receptor potential cation channel subfamily V member 4) in the inter-neuronal transport of calcium and subsequent transmission of itch signals [40,42]. Once activated by histamine, chloroquine, or other potential signaling molecules, these channels cause an influx of calcium ions into peripheral sensory neurons that triggers a biochemical cascade of itch signaling [40,42]. Should the efflux of calcium be impaired in circumstances of calcium pump dysregulation, such as in DD, we hypothesize that the sensation of itch may persist or intensify.

Chronic pruritus is often seen in inflammatory and genetic skin diseases and can lead to the development of psychiatric problems such as anxiety, depression, and social isolation [6,43]. In chronically itchy conditions, calcium signaling dysregulation may contribute to heightened neuronal excitability and continuous activation of itch pathways [41]. Inflammatory dermatoses such as atopic dermatitis and prurigo nodularis often feature elevated levels of interleukin-31 (IL-31), a pruritogenic cytokine [39]. Murine-model studies have shown that IL-31 acts on transient receptor potential (TRP) family receptors to mobilize calcium influx into sensory neurons, making it a potential therapeutic target for treating T-helper-cell-dependent itch [44]. Moreover, chronic neuropathic pruritus often involves maladaptive calcium signaling in affected neurons. The mechanism of GABAergic drugs such as gabapentin, which are often utilized for neuropathic pruritus, involves binding to voltage-gated calcium channels to reduce calcium influx and subsequent release of excitatory neurotransmitters [41]. Studies have also shown that patients with chronic kidney disease have disrupted cutaneous calcium distribution, suggesting a role in the pathogenesis of uremic pruritus [45]. In DD, the mechanism of chronic itch is currently unclear and warrants further investigation.

The psychological distress associated with pruritus may also be related to its physiological mechanisms. Studies have shown that the brain’s response to itch involves areas associated with emotional processing such as the amygdala and temporal cortex, in addition to the sensory cortex [46]. Chronic pruritus stimulates emotion and memory centers, leading to a heightened perception of itch and negative emotions which may be consolidated in the long term [46]. Calcium signaling may play a role in the processing of itch in the central nervous system, given its essential role in synaptic transmission and neuroplasticity that was previously discussed. Dysregulated calcium signaling in circuits between the amygdala, temporal cortex, and sensory cortex may further reinforce the emotional salience of itch, making it more distressing and difficult to ignore.

Pruritus causes an urge to scratch that can be extremely burdensome. While scratching can temporarily relieve discomfort, it often exacerbates what is known as the itch–scratch cycle: a repetitive sequence that can lead to skin damage, secondary infections, and further psychological distress [47]. At the molecular level, scratching activates mechanoreceptors which stimulate calcium influx into sensory neurons [40,48]. This calcium-mediated process may thus paradoxically intensify the sensation of itch, leading to the vicious itch–scratch cycle. Visible skin damage caused by chronic scratching can lead to other dermatologic conditions such as prurigo nodularis [7] and has been shown to cause embarrassment, social withdrawal, and a negative self-image [49]. This further contributes to the psychological impact of pruritus.

Sleep disturbances are another important component of the mind–skin connection. Although the mechanisms behind nocturnal itch are not well understood, the etiology may be related to fluctuations in pruritogenic mediators and skin temperature related to circadian rhythms [50]. In patients with pruritus, the resultant sleep disruptions may contribute to fatigue, irritability, and cognitive impairments that further compound the disease burden of affected individuals [7,50,51]. Numerous studies have documented high rates of sleeplessness in patients with DD [19], supporting the potential connection between pruritus, psychological distress, and sleep disruption. Furthermore, calcium signaling is involved in the regulation of circadian rhythms and sleep–wake cycles [52], suggesting that dysregulated calcium dynamics may contribute to nocturnal pruritus and its associated morbidity. Managing nocturnal pruritus is crucial to DD treatment, as individuals are more susceptible to scratching during sleep, and may wake up with excoriated lesions despite considerable effort to avoid scratching throughout the day. Thus, nocturnal pruritus may fuel the itch–scratch cycle and its associated physical and psychological harms more than daytime pruritus, making it a therapeutic priority.

### 4.4. Clinical Applications and Treatment of Pruritus in Darier Disease

The interplay between pruritus and the psychological burden of DD is highly relevant to patient counseling, requiring multifaceted approaches that go beyond merely treating dermatologic symptoms. Firstly, screening for psychiatric comorbidities and assessment of the severity of pruritus as a routine part of patient care is fundamental, as it will directly impact the navigation of clinical decisions regarding pruritus management [8,9]. Given the large impact of pruritus on the psychological health of patients with DD, among other symptoms such as malodor and pain, the incorporation of patient-reported outcomes into disease severity assessments is crucial. Notably, no consensus on a severity score for DD exists yet.

With regard to clinical interactions, patients should be counseled regarding the genetic basis of the condition. Providers must emphasize that patients’ neuropsychiatric conditions, particularly mood disorders such as depression and anxiety, are not purely sequelae of physical symptoms, but rather a component of the broader phenotype of DD. This understanding can help patients establish more realistic expectations and foster effective coping mechanisms [53], thereby reducing feelings of hopelessness or frustration should their psychological distress persist despite the successful management of dermatologic symptoms.

Regarding treatment for DD, topical corticosteroids, antibacterial skin care practices, and lightweight clothing for reducing physical discomfort are basic strategies of importance [11,54]. However, addressing the psychological distress associated with pruritus requires a multidisciplinary approach that considers both the sensory and emotional dimensions of itch. Advanced biologic therapies, including IL-17 inhibitors, have been shown to effectively reduce itch in many dermatologic conditions [55]. The cytokine profile of DD, which includes increased IL-17 signaling and T helper type 17 (Th17) cells, may suggest a therapeutic target for IL-17 inhibitors [56]. However, immunosuppressive biological therapies should be used with caution in patients with DD who are prone to bacterial and herpetic skin infections [14]. Larger cohort studies are needed to assess the potential safety and efficacy of IL-17 inhibitors in patients with DD. Given the role of opioid imbalance in the mechanism of chronic itch [55], low-dose naltrexone has also been explored as a therapeutic option in DD patients, demonstrating some effectiveness in reducing pruritus [57]. Targeting calcium signaling pathways may be a promising avenue for alleviating itch. TRP channel inhibitors and modulators of calcium-dependent signaling pathways are being explored as potential treatments for chronically itchy conditions [58] and could be applied to DD. Dupilumab, a monoclonal antibody targeting IL-4 and IL-13, has shown excellent efficacy in alleviating pruritus in other dermatologic conditions [59] and may also help manage itch in patients with Darier disease, as evidenced by a recent case report [60]. Psychological interventions such as cognitive behavioral therapy and mindfulness-based approaches, may help patients manage the emotional aspects of itch and break the itch–scratch cycle, thereby reducing psychological distress [61].

From these observations, several key take-home messages for clinicians treating patients with DD emerge: (1) there is a high prevalence of pruritus in DD, as demonstrated in our study and previous reports, which should be assessed as part of management; (2) effective treatment of pruritus among patients with DD requires a comprehensive approach, including screening for the neuropsychiatric comorbidities; and (3) further research is needed to understand the interplay between pruritus and neuropsychiatric conditions in patients with DD.

## 5. Conclusions

In conclusion, patients with DD often report pruritus as a symptom of disease. Therefore, the assessment of pruritus prior to and throughout treatment, using validated scores such as the Numerical Rating Scale for Pruritus, is necessary. Further research is needed to understand the impact of pruritus on patients with DD and the potential role of pruritus in the exacerbation of pre-existing emotional disturbances and genetically predisposed neuropsychiatric comorbidities. When caring for patients with DD, a thorough understanding of each patient’s baseline mental status, alongside the assessment of the additional burden that pruritus or other physical symptoms impose, is essential to effective DD management. We hope that this report will raise awareness and inspire future research into the unique clinical scenario of pruritus among patients with DD.

## Figures and Tables

**Table 1:** Association between proportion of patients reporting symptoms of interest and disease severity.

	Disease Severity (n = 75)			*p* Value ^1^
	Mild n = 25 N (%)	Moderate n = 37 N (%)	Severe n = 13 N (%)	
Itch	21 (84.0)	36 (97.3)	12 (92.3)	0.175
Tingling	9 (36.0)	16 (43.2)	3 (23.1)	0.427
Pain	5 (20.0)	14 (37.8)	7 (53.9)	0.098
Discomfort	11 (44.0)	25 (67.6)	7 (53.9)	0.177
Malodor	3 (12.0)	21 (56.8)	9 (69.2)	<0.001 *
Burning	6 (24.0)	17 (46.0)	2 (15.4)	0.064

^1^ Chi-square test. * Denotes statistical significance.

**Table 2:** Association between proportion of patients reporting symptoms of interest and %BSA affected.

		%BSA Affected (n = 71)				*p* Value ^1^
		N	Mean	STD	Median	
Itch	No	6	12.8	15.6	7.0	0.109
	Yes	66	21.2	14.6	17.0	
Tingling	No	47	20.8	16.3	17.0	0.753
	Yes	25	19.8	11.5	18.0	
Pain	No	47	17.8	12.5	15.0	0.080
	Yes	25	25.6	17.4	20.0	
Discomfort	No	32	18.6	16.5	15.0	0.086
	Yes	40	22.1	13.2	20.0	
Malodor	No	39	14.0	10.1	12.0	<0.001 *
	Yes	33	28.1	15.8	20.0	
Burning	No	48	19.7	15.8	15.0	0.198

^1^ Wilcoxon two-sample test. BSA: body surface area; STD: standard deviation. * Denotes statistical significance.

**Table 3:** Association between patients reporting symptoms of interest and DLQI symptom score.

Patients Who Reported Itch, Tingling, or Pain in the CRF	DLQI Symptom Score, on a Scale of 1 to 4			*p* Value ^1^
	Mean	STD	Median	
No (n = 6)	1.5	0.6	1.5	0.028 *
Yes (n = 68)	2.4	1.0	2.0	

^1^ Wilcoxon two-sample test. DLQI: Dermatology Life Quality Index; CRF: clinical research form; STD: standard deviation. * Denotes statistical significance.

**Table 4:** Association between reported symptoms and neuropsychiatric challenges in patients with Darier disease.

	Itch			Tingling			Pain			Discomfort			Malodor			Burning		
	No N = 7 N (%)	Yes N = 69 N (%)	*p* Value ^1^	No N = 47 N (%)	Yes N = 29 N (%)	*p* Value ^1^	No N = 50 N (%)	Yes N = 26 N (%)	*p* Value ^1^	No N = 32 N (%)	Yes N = 44 N (%)	*p* Value ^1^	No N = 43 N (%)	Yes N = 33 N (%)	*p* Value ^1^	No N = 51 N (%)	Yes N = 25 N (%)	*p* Value ^1^
Anxiety (yes)	1 (14.3)	17 (25.0)	1.000	7 (15.2)	11 (37.9)	0.025	10 (20.0)	8 (32.0)	0.251	4 (12.9)	14 (31.8)	0.059	9 (21.4)	9 (27.3)	0.556	10 (20.0)	8 (32.0)	0.251
Depression (yes)	1 (14.3)	22 (32.8)	0.424	12 (26.7)	11 (37.9)	0.307	14 (28.6)	9 (36.0)	0.514	7 (23.3)	16 (36.4)	0.234	10 (24.4)	13 (39.4)	0.166	12 (24.5)	11 (44.0)	0.086
Suicidal ideation (yes)	1 (14.3)	20 (29.4)	0.665	12 (26.7)	9 (31.0)	0.642	14 (28.6)	7 (28.0)	1.000	8 (25.8)	13 (29.6)	0.723	9 (21.4)	12 (36.4)	0.153	13 (26.0)	8 (32.0)	0.585
Suicide attempt (yes)	1 (14.3)	4 (5.9)	0.396	1 (2.2)	4 (13.8)	0.070	2 (4.0)	3 (12.0)	0.326	1 (3.2)	4 (9.1)	0.397	1 (2.4)	4 (12.1)	0.163	3 (6.0)	2 (8.0)	1.000
Seen a psychiatrist (yes)	0 (0.0)	15 (21.7)	0.333	8 (17.0)	7 (24.1)	0.449	8 (16.0)	7 (26.9)	0.256	4 (12.5)	11 (25.0)	0.176	6 (14.0)	9 (27.3)	0.148	7 (13.7)	8 (32.0)	0.072
Taken psychiatric medication (yes)	0 (0.0)	18 (26.1)	0.188	11 (23.4)	7 (24.1)	0.942	11 (22.0)	7 (26.9)	0.632	5 (15.6)	13 (29.6)	0.159	7 (16.3)	11 (33.3)	0.083	8 (15.7)	10 (40.0)	0.019 **
Previously diagnosed with other psychiatric disorders * (yes)	1 (14.3)	24 (36.4)	0.410	14 (30.4)	11 (40.7)	0.370	14 (29.2)	11 (44.0)	0.205	9 (30.0)	16 (37.2)	0.523	12 (28.6)	13 (41.9)	0.234	13 (26.5)	12 (50.0)	0.047 **

^1^ Chi-square test or Fisher’s exact test. * Including generalized anxiety, obsessive–compulsive disorder, panic disorder, phobias, mood disorders, cyclothymic disorder, bipolar disorder, major depressive disorder, dysthymic disorder, brief psychotic disorders, schizophrenia, schizoaffective, delusional disorder, eating disorders, anorexia nervosa, bulimia nervosa, and/or personality disorders. Missing data points due to non-response are as follows: anxiety (one), depression (two), suicidal ideation (one), suicide attempt (one), previously diagnosed with a psychiatric disorder (three). ** Denotes statistical significance.

## Data Availability

Data are unavailable due to privacy or ethical restrictions.

## Supplementary Materials

The following supporting information can be downloaded at https://www.mdpi.com/article/10.3390/jcm14061818/s1: Supplemental Material S1: clinical report form.
