# Supplementary material for: Pruritus and Neuropsychiatric Symptoms Among Patients with Darier Disease—An Overlooked and Interconnected Challenge
Source: J Clin Med. 2025 Mar 8;14(6):1818. doi: 10.3390/jcm14061818 (PMC11942685; doi:10.3390/jcm14061818)
Supplement: Supplementary file 1 [file jcm-14-01818-s001.zip › jcm-3348794-supplementary.pdf]

<u>Supplementary Material S1</u>

The study of Darier disease
Clinical report form

(A) Personal Information

1. Patient Number: _ _ _
2. Name – initials (first name - surname): _ _

Family information

3. Date of the interview and examination: _ _ / _ _ / _ _
4. Name of the family doctor ____________________________
5. Phone _____________________
6. Name of the personal dermatologist: _____________________
7. Family number: _______
8. Has the patient signed a consent form for medical photography?
    a. Yes, without facial recognition b. Yes, including facial recognition 3. No
9. Pregnancy
    a. Yes b. No

(B) Personal Demographics and Family history

1. Date of birth: _ _ / _ _ / _ _
2. Birth place: 1. Israel 2. Europe 3.North-Africa 4. USA 5. Soviet union
3. Year of Immigration to Israel: _____
4. Ethnic origin: 1. Jewish 2. Muslim 3. Christian- Arab 4. Christian- Non-Arab 5. Druze 6. Circassian
5. Gender: 1. Male 2. Female
6. Marital status: 1. Married 2. Single 3. Widower 4. Divorced 5. Other
7. Number of children: ____
8. Number of brothers: _____
9. Number of sisters:
10. Employment status: 1. Salaried worker 2. Independent worker 3. Unemployed 4. Retired 5. At-home parent
11. Job description: _____________________
12. Years of Education: _____
13. Education: 1. Elementary school 2. High school 3. Secondary education 4. Academic- first degree 5. Academic- Second degree 6. Academic- PhD 7. Other

(C) Personal medical record

1. Current medications: _____________________

    1.1 Do you currently take RITALIN® or other medications from this group?

a) No b) Yes c) I don't know Comments: _______

1.2 If "Yes"- which one? ____________________

1.3 Do you take this medication for Attention deficit hyperactivity disorder?

a) No b) Yes c) I don't know Comments: _______

1.4 Age of drug initation

2. Medical history: _________
3. Previous surgeries: _____________
4. Do you smoke tobacco?

   a) No b) Yesc) I don't know Comments: ______
5. Have you smoked in the past?

   a) No b) Yes c) I don't know Comments: ______
6. Do you drink Alcohol regularly?

   a) No b) Yes c) I don't know Comments: _______
7. Do you use recreational drugs?

   a) No b) Yes c) I don't know Comments: _______
8. Did you use recreational drugs in the past?

   a) No b) Yes c) I don't know Comments: _______

(D) Family history

1. Are there any other people in your family that have Darier disease? I

   a) No b) Yes c) I don't know Comments: _______
2. Are there currently, or have there been in the past, any cases of neurologic diseases in your family?

   a) No b) Yesc) I don't know

   If so, elaborate ____

   2.1 Family relationship:

   a) Father b) Mother c) Brother d) Sister e) Son f) Daughter

   g) Male cousin h) Female cousin i) Uncle j) Aunt

   k) Grandfather l) Grandmother m) Other: ___________________________

   2.2 Does this relative suffer from Darier disease?

   a) No b) Yesc) I don't know
3. Are there currently, or have there been in the past, any cases of mental retardation in your family?

   a) No b) Yesc) I don't know

   If so, elaborate ____

3.1 Family relationship:

b) Father b) Mother c) Brother d) Sister e) Son f) Daughter

g) Male cousin h) Female cousin i) Uncle j) Aunt

k) Grandfather l) Grandmother m) Other: ____________________________

3.2 Does this relative suffer from Darier disease?

a) No b) Yesc) I don’t know

4. Are there currently, or have there been in the past, any cases of psychiatric illnesses in your family?

a) I don’t knowb) Yes No)a

If so, elaborate ____

4,1 Family relationship:

a) Father b) Mother c) Brother d) Sister e) Son

f) Daughter g) Male cousin h) Female cousin i) Uncle j) Aunt

k) Grandfather l) Grandmother m) Other: ____________________________

4,2 Does this relative suffer from Darier disease?

a)No b) Yesc) I don’t know

5. Were there any cases of Suicide in your family?

a) No b) Yesc) I don’t know

If so, elaborate ____

5.1 Family relationship:

Father b) Mother c) Brother d) Sister e) Son f) Daughter

g) Male cousin h) Female cousin i) Uncle j) Aunt

k) Grandfather l) Grandmother m) Other: ____________________________

5.2 Did this relative suffer from Darier disease?

a) No b) Yesc) I don’t know

6. Additional relevant information: _______________

(E) Medical history regarding Darier disease

1. Age at onset of cutaneous manifestations: _____
2. Age of diagnosis? ___
3. Are you currently under medical follow up?

a) No b) Yes c) Other

If so, elaborate ____

3.1 By whom? 1. Community clinic dermatologist 2. Hospital dermatologist 3. Family physician 4. Other: ________

4. Where you ever hospitalized due to Darier disease?
5. What is the area most frequently involved during the last year?

a)Scalp b)Face (forehead, cheeks, peri-oral, chin, eyelids, nasal folds) c) Ears (ear lobes, behind the ears) d) Neck e)Chestf) Back

h) Abdomen i) Body folds (axilla, groin, abdomen folds, elbow folds, knee folds, breast folds) j)Palms k)Feet l)Mucosa (Oral, genital)

m) Buttocks n) Upper extremities o) Lower extremities p) Nails . Other: _______________

6. Which symptoms do you experience as part of your Darier's disease?

a) Itch b) Tingling c) Pain d) Discomfort e) Malodor f) Burning g) Other: ________

(F) Neurological and mental symptoms

1. Do you currently, or did you have in the past neurological complaints?

1.1 age of onset: _________

1.2 Is it active in the last year?

a) No b) Yes c) I don't know Comments: _________

2. Do you currently, or did you have in the past from Epilepsy?

2.1 age of onset: _________

2.2 Is it active in the last year?

3. a) No b) Yes c) I don't know Comments: _________

Do you currently, or did you suffer in the past from chronic headaches?

3.1 age of onset: _________

3.2 Is it active in the last year?

a) No b) Yes c) I don't know Comments: _________

4. Do you currently, or did you suffer in the past from migraines?

4.1 age of onset: _________

4.2 Is it active in the last year? _____

a) No b) Yes c) I don't know Comments: _________

5. Do you currently, or did you suffer in the past from memory impairment?

5.1 age of onset: _________

5.2 Is it active in the last year?

a) No b) Yes c) I don't know Comments: _________

6. Do you currently, or did you suffer in the past from anxiety?

6.1 age of onset: _________

6.2 Is it active in the last year? ________

7. Do you currently, or did you suffer in the past from major depression?

7.1 age of onset: __________

7.2 Is it active in the last year?

a) No b) Yes c) I don’t know Comments: __________

8. Do you currently, or did you have in the past any suicidal ideation?

8.1 age of onset: __________

8.2 Is it active in the last year?

a) No b) Yes c) I don’t know Comments: __________

9. Did you ever try to commit suicide?

9.1 Age: ______

9.2 11.2 Any attempt to commit suicide in the last year:

a) No b) Yes c) I don’t know Comments: __________

10. Do you currently, or did you have in the past difficulty with restraining violent behavior?

12.1 Age of onset: ______

12.2 Is it active in the last year?

a) No b) Yes c) I don’t know Comments: __________

11. Did you every seek psychological counseling due to mental distress caused by Darier disease?

a) No b) Yes c) I don’t know Comments: __________

16.1 At what age: _______

16.2 Did you seek psychological counseling in the past year? ____

12. Did you every seek psychiatric counseling?

a) No b) Yes c) I don’t know Comments: _________

17.1 At what age: _______

17.2 Did you seek psychiatric counseling in the past year? _____

13. Did you ever use mental health medications?

a) No b) Yes c) I don’t know Comments: __________

14. Is there any other relevant information regarding mental/neurological complaints?

a) No b) Yes c) I don’t know Comments: __________

15. Do you currently, or did you have suffer in the past from psychiatric illness?

a) No b) Yes c) I don’t know Comments: __________

15.1 What as your diagnosis?

a) Anxiety disorder 1.1 Generalized 1.2 Obsessive-Compulsive disorder 1.3 Panic disorder 1.4 Phobias 1.5 Other 2

b) Mood disorder 2.1 Cyclothymic 2.2 Bipolar 2.3 Major depression 2.4 Dysthymic

disorder 2.5 Other

c) Psychotic disorder 3.1 Brief psychotic episode 3.2 Schizophrenia 3.3 Schizoaffective 3.4 Delusional disorder 3.5 Other

d) Eating disorder 4.1 Anorexia nervosa 4.2 Bulimia nervosa 4.3 Other5.

e) Personality disorder? 5.1 Borderline 5.2 Antisocial 3. Other

f) Other- please elaborate

g) I don’t know the exact diagnosis

16. 12.2 Is it active in the last year?

a) No b) Yes c) I don’t know Comments: _________

(G) Systemic treatments

What systemic treatments did you use during your disease?

1. Acetretin (e.g. Neotigason):

a) No b) Yes c) I don’t know Comments: _________

1.1 age at time of first treatment: _____

2. Isotertinoin (e.g. Roaccutane):

a) No b) Yes c) I don’t know Comments: _________

2.1 age at time of first treatment: _____

3, Systemic antibiotics:

a) No b) Yes c) I don’t know Comments: _________

3.1 age at time of first treatment: _____

4. Systemic steroids:

a) No b) Yes c) I don’t know Comments: _________

4.1 age at time of first treatment: _____

5. Others:

a) No b) Yes c) I don’t know Comments: _________

5.1 age at time of first treatment: _____

Doctor: ____

Date: _ _ / _ _ / _ _
